# Editorial: Artificial intelligence for smart health: learning, simulation, and optimization

**DOI:** 10.3389/fphys.2024.1541057

**Published:** 2024-12-23

**Authors:** Bing Yao, Nathan Gaw, Hyo Kyung Lee

**Affiliations:** ^1^ Department of Industrial and Systems Engineering, The University of Tennessee, Knoxville, TN, United States; ^2^ Department of Operational Sciences, Air Force Institute of Technology, Wright-Patterson AFB, OH, United States; ^3^ School of Industrial Management and Engineering, Korea University, Seoul, Republic of Korea

**Keywords:** machine learning and AI, health informatics, simulation optimization, smart health, data mining

With rapid developments in medical sensing and imaging, we now live in an era of data explosion in which large amounts of data are readily available in clinical environments. The fast-growing biomedical and healthcare data provide unprecedented opportunities for data-driven scientific knowledge discovery and clinical decision support. Our Research Topic aims to catalyze synergies among biomedical informatics, machine learning, computer simulation, operations research, systems engineering, and other related fields with three specific goals: (1) develop cutting-edge data-driven models to accelerate scientific knowledge discovery in biomedicine using healthcare data collected from laboratory systems, imaging systems, and medical and sensing devices; (2) develop advanced simulation and calibration algorithms to build personalized digital twins by effectively assimilating patient-specific medical data with population-level computer models, facilitating precision medical planning; (3) develop innovative optimization algorithms for optimal medical decision making in the face of uncertainty factors, conflicting objectives, and complex trade-offs. This Research Topic, containing 10 articles, will offer a timely collection of information to benefit researchers and practitioners working in the broad fields of biomedical informatics, healthcare data analytics, medical image processing, and health-related AI.

Jiang et al. investigated the development and implementation of a high-fidelity simulation training course for fostering medical and nursing collaboration in China, guided by the Fink integrated curriculum design model. This training course was delivered to 14 nursing students and 8 clinical medicine students between March and July 2022. The results showed high satisfaction, increased self-confidence, and positive evaluations across various teaching practice dimensions. The study underscores the value of standardized simulation curricula in advancing healthcare education in China.

Rovati et al. evaluated the usability, workload, and acceptance of a digital twin application designed to simulate patient clinical trajectories based on EHR data for critical care education. Tested with 35 first-year internal medicine residents, the application demonstrated good usability and low to moderate workload. Residents expressed interest in using the digital twin application for ICU training and suggested improvements in clinical fidelity, interface design, learning experience, gaming elements, and implementation strategies.

Xie et al. developed a multi-branching ResNet model for atrial fibrillation detection from single-lead ECG signals. This method combines continuous wavelet transform for feature extraction with a multi-branching architecture to handle class imbalance in ECG datasets. Their framework was evaluated on two databases: PhysioNet/CinC challenge 2017 and private datasets from the University of Oklahoma Health Sciences Center. Their model achieved F1 scores of 0.8865 and 0.7369 on the two datasets respectively, demonstrating strong performance in balancing precision and recall.

Patharka et al. provided a systematic review of research challenges in modeling biomedical temporal data, including missing values, capturing multi-dimensional correlations, and accounting for short- and long-term temporal patterns. This paper categorizes time series models into statistical, machine learning, and deep learning approaches, and further discusses their strengths and limitations. Strategies such as model enhancement, ensemble forecasting, and hierarchical models are examined for improving clinical predictions. It also explores implementation challenges in biomedical data modeling and outlines future directions for integrating AI in healthcare.

Kim et al. developed a Timely Early Warning System for Septic Shock (TEW3S), which emphasizes predicting the onset timing of septic shock to assist proactive clinical interventions. Utilizing machine learning and EHRs from the MIMIC-IV database, TEW3S achieved 94% accuracy in predicting all shock events with a maximum lead time of 8 h. By addressing the limitations of traditional risk-based prediction systems, this approach highlights the critical role of timeliness in improving patient outcomes during acute deterioration in hospital settings.

Rao et al. developed a multi-scale long short-term memory (LSTM) neural network trained with a variety of time scale data for classifying fetal heart rate patterns during labor. They employed preprocessing techniques to mitigate negative effects such as missing signals and artifacts on the model, and further utilized data augmentation techniques to address the data imbalance issue. Their framework was evaluated on the CTU-UHB dataset and achieved superior performance compared with traditional LSTM.

Stanik et al. developed a predictive model to identify stroke survivors at high risk of seizures following an infection, using data from the Long-Term Care Minimum Data Set. Data balancing techniques and feature selection methods are incorporated into machine learning models (Logistic Regression, Random Forest, XGBoost, Neural Network), achieving high accuracy in seizure prediction. Key factors contributing to seizure risk identified by this article included therapy hours, independence in daily activities, and mood.

Trevena et al. developed a graph-based patient simulation application designed to model critically ill patients with sepsis. The authors utilize directed acyclic graphs to represent the complex physiological and medication interactions during the first 6 h of critical illness. Their system consists of three core components: a cross-platform frontend for clinicians and trainees, a cloud-hosted simulation engine, and a graph database to determine the progression of each simulation. The simulation architecture demonstrates the potential to help train future generations of healthcare professionals and facilitate clinicians’ bedside decision-making.

Wang et al. developed a three-phase methodology for emotion recognition from electroencephalography signals. Their framework addresses the challenges of capturing the complex, nonlinear, and nonstationary dynamics of brain activity by integrating manifold embedding, multilevel heterogeneous recurrence analysis, and ensemble learning. Evaluated on the SJTU-SEED IV database, their method demonstrates superior performance compared to existing commonly used techniques.

Meyers et al. investigated the sources of variability affecting operating room (OR) efficiency. The OR process was segmented into eight stages to quantify key process times, such as procedure duration and start time delay. The authors developed linear mixed models to evaluate the effects of factors such as the primary surgeon, anesthesia provider, and procedure type on OR efficiency. This study emphasizes the importance of segmenting the OR process into finer stages for better understanding of efficiency.

Finally, we extend our sincere gratitude to the reviewers for their thoughtful and constructive feedback on the manuscripts submitted to this Research Topic. Their insightful evaluations have significantly contributed to enhancing the quality and impact of this Research Topic.

